# Evaluating TcAs for Use in Biotechnology Applications

**DOI:** 10.3390/biotech14010005

**Published:** 2025-01-25

**Authors:** Cole L. Martin, John H. Hill, Brian D. Wright, Solana R. Fernandez, Aubrey L. Miller, Karina J. Yoon, Suzanne E. Lapi, Stephen G. Aller

**Affiliations:** 1 Graduate Biomedical Sciences Pathobiology, Physiology and Pharmacology Theme, University of Alabama at Birmingham, Birmingham, AL 35294, USA; martin94@uab.edu; 2 Department of Biochemistry & Molecular Genetics, University of Alabama at Birmingham, Birmingham, AL 35294, USA; jhh1492@uab.edu; 3 Center for Integrative Structural Biology, University of Alabama at Birmingham, Birmingham, AL 35205, USA; 4 Graduate Biomedical Sciences Biochemistry & Structural Biology Theme, University of Alabama at Birmingham, Birmingham, AL 35294, USA; 5 Department of Radiology, University of Alabama at Birmingham, Birmingham, AL 35294, USA; brianwright@uabmc.edu (B.D.W.); sfernandez@uabmc.edu (S.R.F.); lapi@uab.edu (S.E.L.); 6 Department of Cell, Developmental & Integrative Biology, University of Alabama at Birmingham, Birmingham, AL 35294, USA; aubrey44@uab.edu (A.L.M.); kyoon@uab.edu (K.J.Y.)

**Keywords:** ABC toxin complexes, XptA2, protein engineering, Cryo-EM, protein dynamics, positron emission tomography

## Abstract

ABC toxin complexes (Tcs) are tripartite complexes that come together to form nano-syringe-like translocation systems. ABC Tcs are often compared with *Bacillus thuringiensis* (Bt) toxins, and as such, they have been highly studied as a potential novel pesticide to combat growing insect resistance. Moreover, it is possible to substitute the cytotoxic hypervariable region with alternative peptides, which promise potential use as a novel peptide delivery system. These toxins possess the unique ability to form active chimeric holotoxins across species and display the capability to translocate a variety of payloads across membrane bilayers. Additionally, mutagenesis on the linker region and the receptor binding domains (RBDs) show that mutations do not inherently cause a loss of functionality for translocation. For these reasons, Tcs have emerged as an ideal candidate for targeted protein engineering. However, elucidation of the specific function of each RBD in relation to target receptor recognition currently limits the use of a rational design approach with any ABC Tc. Additionally, there is a distinct lack of targeting and biodistribution data for many Tcs among mammals and mammalian cell lines. Here, we outline two separate strategies for modifying the targeting capabilities of the A subunit (TcA) from *Xenorhabdus nematophilus*, Xn-XptA2. We identify novel structural differences that make Xn-XptA2 different than other characterized TcAs and display the modular capabilities of substituting RBDs from alternative TcAs into the Xn-XptA2 scaffold. Finally, we show the first, to our knowledge, biodistribution data of any TcA in mice.

## 1. Introduction

ABC toxin complexes (Tcs) are a class of α-pore forming toxins (αPFTs) that consist of three subunits capable of assembling into a single protein apparatus with the ability to shuttle and translocate toxic peptides into target cells [1,2,3]. ABC Tcs are naturally secreted in bacteria and target various organisms as diverse as plants, insects, and humans [3,4,5]. Some of these bacteria live symbiotically with entomopathogenic nematodes and target a broad range of insects for both bacterial and nematode sustenance and proliferation [3,6,7,8]. *Xenorhabdus nematophilus* is an ABC Tc-producing bacterium that lives symbiotically with *Steinernema carpocapsae* nematodes [9,10,11]. Once *S. carpocapsae* invades target larvae such as *Helicoverpa zea* (corn earworm), *X. nematophilla* are released into the insect hemocoel or ingested into the insect midgut [11]. After invasion of the insect host, *X. nematophillus* releases insecticidal ABC Tcs causing cytotoxicity resulting in the death of the organism and proliferation and reproduction of the bacterium and nematode symbionts [11].

The Tcs released in *X. nematophillus* contain a mixture of two A subunits (TcAs), a single B subunit (TcB), and a single C subunit (TcC). Each TcA displays the capability to form independent tripartite complexes with the TcB and TcC subunits. The two TcAs termed Xn-XptA1 and Xn-XptA2 are the targeting components of each complex and display variations in insect specificity. Xn-XptA1 displays a broad range of insecticidal activity while Xn-XptA2 displays a narrow range of insecticidal activity, indicating alternative mechanisms of the host tropism [10,11].

The TcAs are the largest subunits of the complex and contain a translocation channel with hydrophobic loops at the end allowing for membrane permeation [12,13,14,15]. The TcB and TcC subunits form a tight complex after translation and are responsible for shuttling the toxic peptide internally until it binds to the TcA [16,17]. The toxic peptide is an ADP-ribosyltransferase with the potential to polymerize actin to kill the target cell [18,19,20]. Once bound to the TcA, the TcB forms a gate that allows for the ADP-ribosyltransferase to thread into the TcA translocation channel [19]. Upon TcA binding and an environmental pH change, the complex undergoes a conformational change into an active state in which the translocation channel is exposed and pierces the target cell membrane [13,21]. After membrane permeation, the ADP-ribosyltransferase is linearized and transferred into the target cell where it subsequently refolds, resulting in actin polymerization and cell death [14,17].

Due to the unique mechanism of ABC toxins, studies have explored the possibility of utilizing this class of proteins as intracellular protein shuttles [22,23]. These studies conclude that substitutions for the autoproteolytically cleaved toxic peptide can be made if the substituted peptide remains within certain biochemical and size-based parameters [22]. Although these landmark results suggest that alternative peptides can be transported across cell membranes by modifying the TcC subunit, how to alter the targeting specificity of the TcA subunit is still unclear. Furthermore, there is still uncertainty about which receptors are targeted by different TcA homologs, as well as a lack of a distinct toxicity profile among mammalian organisms.

In this manuscript, we report the use of two strategies to alter targeting specificity in Xn-XptA2 using genetic insertions of the pharmacophore sequence of DOTATATE, which is contained within the four amino acids of the TATE group (Tyrosine, Tryptophan, Lysine, and Threonine) as well as a full receptor binding domain (RBD) substitution between *Xenorhabdus nematophilus* (Xn)-XptA2 and *Photorhabdus luminescens* (Pl)-TcdA1. We analyze the results of these strategies through binding and toxicity assays as well as structural analyses. Xn-XptA2 maintains structural integrity after minor and even some major alterations of the outer shell of its TcA. However, modification of the outer loops of the neuraminidase domains (NDs) causes the structure to be locked into an alternative, State 2 conformation, indicating that these loops are important for conformational dynamics. Furthermore, we report the toxicity profile of Xn-XptA2 Tc and Pl-TcdA1 Tc on two separate pancreatic cancer cell lines (Panc1 and MiaPaCa2) in comparison to the Xn-XptA2 RBD chimera Tc. Lastly, we report the first biodistribution data for Xn-XptA2 in mice, which is, to our knowledge, the first biodistribution data for any TcA in mammals.

## 2. Materials and Methods

### 2.1. Constructs

The full-length Xn-XptA2 wt DNA (WP_041979038.1.) was subcloned into the pET24a vector (Novagen, Madison, WI, USA) with a C-terminal 6× Histidine tag. The Xn-XptA2 YWKT construct was synthesized by ligating the YWK sequence into the Xn-XptA2 wt pET24a vector between base pairs 3543 and 3549 after digesting with BamHI and XhoI (NE Biolabs, Ipswich, MA, USA) and an additional CIP digest reaction (NE Biolabs). The duplicate reactions were DNA precipitated using 10% volume mix of potassium acetate and 2.5× total volume mixture of 100% ethanol. After precipitation, we pelleted the DNA, washed it with 70% ethanol by vortexing the pellet, and pelleted the DNA once again before decanting the supernatant and drying the pellet for 1 h in the fume hood. Once dry, the pellet was resuspended in 10 mM Tris buffer and transformed into One Shot TOP10 Chemically Competent *E. coli* cells (Thermo Fisher Scientific, Waltham, MA, USA) and plated on Kanamycin-supplemented agar plates. Colonies were picked and grown in 10 mL LB/Kanamycin cultures overnight, plasmid isolation was performed on each culture, and the sequence was verified through comprehensive sequence analysis. Once verified, this construct was used as a template to mutate the final residue in the YWKT chimera (D1182) starting at codon 3546 through a mutagenesis point mutation reaction. The sequence was verified after DNA isolation, as previously described. These plasmids were transformed into BL21 (DE3) competent cells (Thermo Fisher Scientific, Waltham, MA, USA), and colonies were selected from plates containing Kanamycin for selectivity. The Xn-XptA2 RBD C chimera was synthesized by the addition of two restriction enzyme cut sites into the wt sequence at codon 4977 (NarI) and 5361 (PasI) flanking the DNA encoding for Xn-XptA2 RBD C. This was achieved through mutagenesis reactions and resulted in silent mutations for the RE cut sites verified through sequencing analysis. The sequence containing Pl-TcdA1 RBD D was ordered (IDT) with NarI and PasI RE cut sites flanking the RBD sequence. The native Xn-XptA2 RBD C was digested with PasI and NarI to remove the native RBD C DNA, and the same process was utilized to isolate the Pl-TcdA1 RBD D insert DNA. Once isolated, these fragments were both ligated back into the pET24a vector and verified through kanamycin selective transformations and sequencing analysis.

### 2.2. Protein Expression and Purification

For cryo-EM, unlabeled recombinant wild type (wt) Xn-XptA2, Xn-XptA2 YWKT, and Xn-XptA2 RBD chimera were expressed in *Escherichia coli* BL21(DE3) competent cells (Thermo Fisher Scientific, Waltham, MA, USA). Cells were grown in Luria Broth (LB) to an OD_600_ of 0.6, chilled to 16 °C, and induced with 1 mM isopropyl-β-D-thiogalactoside (IPTG) (Thermo Fisher Scientific, Waltham, MA, USA) for 18 h. The cells were then harvested and resuspended in lysis buffer containing 20 mM Tris pH 7.5, 100 mM NaCl, 20 mM Imidazole, 0.05% Sodium Azide, and protease inhibitor cocktail (Sigma-Aldrich, Burlington, MA, USA) and lysed using a cell disruptor (Constant Cell Disruption Systems, Daventry, UK) at 15 psi. After centrifugation at 38,400× *g* for 45 min to remove cell debris, the supernatant was collected and applied to a pre-equilibrated (20 mM Tris pH 7.5, 100 mM NaCl, 20 mM Imidazole, 0.05% Sodium Azide, and protease inhibitor cocktail) nickel affinity AP-2 column (Waters, Milford, MA, USA) at 4 °C, eluting in 20 mM Tris pH 7.5, 100 mM NaCl, 300 mM Imidazole, 0.05% Sodium Azide, and protease inhibitor cocktail. The eluate was concentrated with a 100 kDa cutoff concentrator (Millipore, Burlington, MA, USA) and ultracentrifuged at 217,000× *g* for 30 min at 4 °C in a TLA 120.2 (Beckman Coulter, Brea, CA, USA) rotor. The supernatant was applied to a HiPrep 16/60 Sephacryl S-400 HR column pre-equilibrated with 100 mM NaCl and 10 mM Tris pH7.5. SDS PAGE was utilized to analyze XptA2 purity within the eluted fractions and the pure fractions were concentrated using a 100 kDa cutoff concentrator (Millipore) and used for further structural analyses.

### 2.3. Protein Conjugation and Radiolabeling

The conjugation of DFO to Xn-XptA2 or Xn-XptA2 YWKT was performed similarly to previous methods [24,25,26,27,28]. The protein solutions were adjusted to pH 9 using sodium carbonate buffer and combined with between two and eightfold molar excess of deferoxamine-Bz-SCN (DFO) in DMSO. The resulting mixture was incubated at 37 °C for 1 h with agitation. After incubation, the unreacted DFO was removed using a 40 k MWCO Zeba spin column preconditioned with 1 M HEPES buffer (pH 7). Concentration of the conjugate was accessed via the BCA assay prior to labeling.

Zirconium-89 was produced in the University of Alabama at Birmingham cyclotron facility, diluted with 1 M HEPES buffer, and neutralized with 2 M sodium hydroxide [29]. Xn-XptA2-DFO or Xn-XptA2 YWKT-DFO were combined with the neutralized Zirconium-89 solution in ratios of 1:74 mg:MBq (1 mg:2 mCi) and incubated for 1 h at 37 °C with agitation. After incubation, the labeled protein cluster was challenged with a 50 mM DTPA solution, and the radiochemical purity was checked via iTLC using a 50 mM DTPA solution as the running buffer.

### 2.4. In Vitro Cell Studies

The Lindmo assay was used to determine the in vitro binding characteristics of the modified proteins. Concentrations ranging from 0.5 to 5 × 10^6^ cells/mL of AR42J (SSTR2+) cells (CRL-1492, ATCC, Manassas, VA, USA) were combined with 50 µL of a 1% BSA solution containing 5 nCi of the radiolabeled proteins. The cells were allowed to incubate at room temperature with gentle agitation for 1 h, pelleted, and washed with cold PBS three times. The total amount of activity was counted, and the binding was calculated as the ratio of bound activity to the total amount of activity.

Cell viability assays were performed as previously reported [30,31]. Briefly, Panc1 (CRL-1469, ATCC, USA) and MiaPaCa2 (CRL-1420, ATCC, USA) cells were plated in 96-well tissue culture treated plates and allowed to adhere overnight in 1× DMEM (Corning Inc., Corning, NY, USA) #15-013-CM), FBS (R&D Systems, Minneapolis, MN, USA, S11150H) to a final concentration of 10%, and 200 mM L-glutamine (Gibco 25030-081) to a final concentration of 2 mM at 37 °C and 5% CO_2_. Serial dilutions of each protein complex were prepared (0.01–250 ug/mL) and added to the wells for 96 h. AlamarBlue reagent (Thermo Fisher Scientific, Waltham, MA, USA) was added to the wells according to the manufacturer’s instructions (Thermo Fisher Scientific, Waltham, MA, USA). Absorbance was read on a Victor x5 microplate reader (Perkin Elmer, Waltham, MA, USA) at 590 nm. Data are presented as the mean ± SEM of one or three independent experiments. Each individual experiment has 4 replicates for each concentration of protein, with an average of 12 replicates (4 replicates × 3 experiments). Two controls were added for each experiment. The first control contains a row of wells that only has media in it with no cells (blank). These blanks had AlamarBlue added, and the absorbance was subtracted out of the rest of the plate to determine the level of background. One row of wells contains cells but did not receive any dose of protein and was subsequently treated with protein buffer. This is the row that was used to normalize all values.

### 2.5. In Vivo Studies

All animal experiments were conducted according to the guidelines of the Institutional Animal Care and Use Committee (IACUC-21445) and approved by the University of Alabama at Birmingham Animal Studies Committee. All PET imaging and biodistribution studies were performed using Balb/C mice from Charles River Laboratories. The mice were injected via tail vein with 100 µL of 3.5 MBq (50 µg, 100 µCi) of [^89^Zr] Xn-XptA2 in saline. PET (energy window 350–650 keV) and CT (voltage 80 kVp, current 150 µA, 720 projections, scan time 5 min) images were acquired using a GNEXT small animal PET/CT scanner (Sofie Biosciences, Dulles, VA, USA) at 1, 2, 5, and 7 days after injection. After 7 days post-injection, mice were euthanized, and organs were harvested, weighed, and assayed in the gamma counter for biodistribution studies. The PET images were reconstructed using a 3D-OSEM (Ordered Subset Expectation Maximization) algorithm (24 subsets and 3 iterations) with random, attenuation, and decay correction. The CT images were reconstructed using a Modified Feldkamp Algorithm. Image analysis was performed using the Vivoquant software suite (Invicro, Needham, MA, USA). Radioactivity associated with each organ was expressed as a percentage of the injected dose per gram of organ (%ID/g).

### 2.6. Single Particle Cryo-Electron Microscopy

#### 2.6.1. Cryo-EM Grid Preparation

For the high-resolution structure of Xn-XptA2 State 2, Xn-XptA2 YWKT, and Xn-XptA2 RBD chimera, cryo-EM grids were prepared using a Vitrobot Mark IV (Thermo Fisher). Four microliters of each protein at 1.5 mg/mL for each sample (10 mM Tris, 100 mM NaCl, pH 7.5) were applied to a glow-discharged Quantifoil holey carbon grid (R2/2 and R2/1), blotted for 4 s at a blot force of 4 before plunge freezing into liquid ethane. Grids were stored in liquid nitrogen prior to shipping for screening and data collection.

#### 2.6.2. Cryo-EM Imaging

For the high-resolution Xn-XptA2 wt State 2 map, data were collected using the Vanderbilt University Technai T30 Polara (FEI) microscope equipped with a Gatan K2 Summit DED in counting mode. A total of 1605 movies (50 frames per movie) were collected with defocus ranging from 0.8 µm to 2.5 µm and a total dose of 75 e^−^/A^2^.

For the high-resolution Xn-XptA2 YWKT State 2 map, data were collected at the Baylor School of Medicine using a Jeol JEM3200 microscope (JEOL Ltd., Akishima, Tokyo, Japan) equipped with a Gatan K2 Summit camera. A total of 636 movies at 60,000× magnification (pixel size 0.64 Å) were collected using an accelerating voltage of 300 kV, a spherical aberration of 2.7 mm, and a total exposure dose (e^−^/Å^2^) of 10.

For the high-resolution Xn-XptA2 RBD C chimera map, data were collected at the Ohio State University Center for Electron Microscopy and Analysis (CEMAS) using a Thermo Fisher Titan Krios microscope equipped with a Gatan K3 camera. A total of 2213 movies were collected at 89,000× magnification and with pixel size 0.89 Å using an accelerating voltage of 300 kV, a spherical aberration of 2.7 mm, and a total exposure dose (e^−^/Å^2^) of 35.

#### 2.6.3. Cryo-EM Data Processing

Refinement of the 4.3 Å Xn-XptA2 YWKT construct of the A-subunit of *Xenorhabdus nematophila* was accomplished using CryoSPARC. (ver 4.5.3). A total of 636 movies were imported at pixel size 0.642 (J5). The resulting 636 micrographs were then patch motion corrected (J6). Curated exposure was run, resulting in 584 micrographs accepted (J9). Blob picking was then performed with minimum particle diameter of 100 Å and maximum diameter of 300 Å in conjunction with both elliptical and circular blobs and 300 targeted peaks (local maxima) (J14). This resulted in 65,922 particles extracted (113 particles/micrograph) with an extracted box size of 778 pixels and a Fourier crop to box size of 258 pixels, which was subjected to 2D classification in 50 classes. Five classes showing clear views of the balloon, like TcA from alternate viewpoints, were selected for use in template picking against the accepted exposures (J12) with a particle diameter of 280 Å and 2000 targeted peaks. The resulting picks were extracted using box size of 778 pixels and a Fourier crop to box size of 258 pixels and subjected to 2D classification in 100 classes (J30). This resulted in 19 classes with proper symmetrical A subunits. These classes were used as inputs for a 2-fold class run of ab initio using C1 symmetry (J32). These particles were further subjected to non-uniform refinement (J36). The 90,703 particles were used in 3D classification with input initialization mode, filter resolution 4Å, C5 symmetry, convergence criterion 0.5%, and two classes selected (J38). The class having the best-defined pentameric shape was selected, and subsequent Homo refinement using the output refined volume and static mask yielded a 4.3 Å map that was deemed optimal for further atomic modeling (J40).

Refinement of the TcdA1 RBD4 Chimera data set was accomplished using CryoSPARC (ver 4.5.3). A total of 2213 movies were imported at pixel size 0.4495 with the gain reference flip/defect file in Y option set to true (J1). The resulting 2213 micrographs were then patch motion corrected with 222 movies used in the denoiser training data (J3). Curate exposures were then completed with default settings, resulting in 2082 (94%) exposures accepted, which were subsequently placed through patch CTF correction and an additional round of curate exposures, resulting in a final of 1996 exposures accepted (J6). The exposure sets tool was then used with split randomize set to true, split number of batches set to 1, and split batch size set to 200 (J8). Blob picking was then performed on 200 micrographs with minimum particle diameter of 100 and maximum diameter of 300 alongside use of an elliptical blob focused on 100 targeted peaks (local maxima) (J9). Then, ~8846 particles were extracted with an extraction box size of 1332 pixels and a Fourier crop to box size of 332 pixels, with the results being saved in 16-bit floating points (J10). These particles were then subjected to 2D classification for 50 classes in the absence of a circular mask (J11). The 5 classes showing the most defined stem regions (while maintaining acceptable symmetry) of the A-subunit were used in template picking alongside the curated exposures from J6 with a particle diameter of 300 and 100 targeted peaks (J13). The resulting picks were extracted from the same 1996 micrographs with a box size of 1112 pixels and saved in 16-bit floating point (J14). A second round of 2D classification was then completed using 50 classes and without the use of a circular mask imposed onto 2D classes. The best 17 classes were then selected for use as templates in subsequent micrograph extraction with extraction box size of 1112 pixels and Fourier box size of 370 pixels. The update location and update alignment options are set to true, and the alignment 2D field is used for re-centering (J20). Additionally, an ab initio initial model job was run using C5 symmetry to generate a map, which was paired with the extracted micrographs from J20 and used in non-uniform refinement with C5 symmetry, yielding a final resolution of 3.39 angstroms.

Refinement of the 3.9 Å Xn-XptA2 State 2 structure was accomplished using Relion 3.1.2. A total of 1605 movies were imported at pixel size 1.268 (J1). CTFfind was then completed using micrographs without dose weighting and the astigmatism was set to 100 Å (J2). Auto-picking was completed with C5 symmetry and using Laplacian-of-Gaussian setting. This resulted in 26,498 particles over 100 micrographs (J7). Particle extraction was run to re-extract refined particles (J8). Two-dimensional classification was then performed, with the subsequent classes selected from model.star file and then used for an additional round of auto-picking, resulting in 29,096 particles over 100 total micrographs (291 particles/micrograph) (J11). These particles were then extracted with a box size of 354 pixels, and the particles were not rescaled (J12). A further round of 2D classification was completed, generating 70 classes with a regularization parameter of 2, 25 iterations completed, and using a mask diameter of 496 Å (J13). The best classes were selected (J14), used in auto-picking again (330 Particles/Micrograph) (J16), and extracted using default parameters. (J17) An additional round of 2D classification was then performed using the same parameters from Job 11. This cycle of generating 2D classes for use in picking was repeated several times, each time lowering the diameter of the mask used until enough 2D classes with optimal unique views could be selected for use in downstream 3D classification jobs (J38). Three-dimensional classification was used with a regularization parameter set to 4 and 25 iterations completed with a mask diameter of 320 Å and image alignment performed with an angular sampling interval of 7.5 degrees, as well as allowing coarser sampling (J44). The best 3D classes were subsequently selected (J45) and then refined using solvent-flattened FSCs and a mask diameter of 320 Å (J46). A mask was then created with lowpass filter set to 15 Å, an initial binary threshold of 0.00222, and a binary extension of 3 pixels (J47). A final extraction was performed on the initial inputs for the input CTFfind job and was refined against the refined particles star file from job 45 (J87). Three-dimensional classification was performed on the output with 10 classes, 50 iterations, and a mask diameter of 320 Å with coarser sampling allowed and image alignment enabled (89). These classes were selected and then re-refined as previously detailed, following mask generation (J92). Post-processing of the refined 3D classes and generated mask was then completed with the lowest resolution for B-fit being set to 10 Å and estimate B-factor automatically set to true. The selected classes from job 90, refined classes from job 91, and mask created in job 92 were all used as inputs for 3D auto-refine, with a new mask being generated in the preceding job. Finally, another post-processing was completed with the same parameters and using refined J95 classes and the mask created in job 96 (J97).

## 3. Results

### 3.1. Analyses of Pharmacophore Insertion Within Xn-XptA2

To optimize the natural peptide shuttling and translocation mechanism of Xn-XptA2, our first strategy consisted of inserting the pharmacophore sequence of the TATE region of the DOTATATE peptide into the outermost loops of the NDs to alter the targeting potential of Xn-XptA2. TATE is the somatostatin analog Tyr3-octreotate and targets somatostatin receptors (SSTRs). It has a high affinity to somatostatin receptor 2 (SSTR2) [32]. TATE is FDA-approved as an imaging agent (when labeled with ^68^Ga and ^64^Cu—DETECTNET) and treatment (when labeled with ^177^Lu) for patients with SSTR-positive neuroendocrine tumors (NETs) [33,34,35]. SSTRs are highly expressed in various NETs, including pancreatic tumors, lung tumors, and neuroblastomas. They are expressed in low to moderate in other organs throughout the body [34].

**Figure 1:**
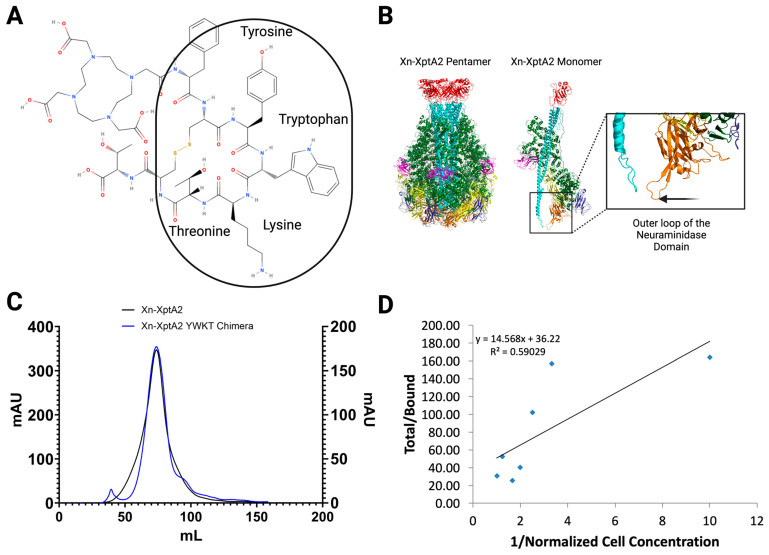
Analyses of the YWKT pharmacophore insertion into the Xn-XptA2 construct. (**A**) The 2D structural representation of the DOTATATE peptide with the 4 amino acid pharmacophore sequence (YWKT) circled. (**B**) Xn-XptA2 wt structure (8TQE) colored by domain displaying both a pentameric and monomeric view with a zoomed-in view of the outer loops of the neuraminidase domain. (**C**) Size exclusion chromatography graphs comparing Xn-XptA2 to the Xn-XptA2 YWKT chimera. (**D**) Lindmo binding assay results from the Xn-XptA2 YWKT construct after DFO conjugation using 8-fold molar excess DFO and Zirconium-89 radiolabeling.

We examined the pharmacophore of TATE and found that the binding properties of the peptide reside within a four-amino acid sequence on the peptide: Tyrosine (Y), Tryptophan (W), Lysine (K), and Threonine (T) (Figure 1A). Previous studies suggest that the NDs, together with the RBDs, contribute to the host tropism of the TcA [14,36]. After careful examination of the outer shell of Xn-XptA2, we decided the most appropriate location for the insertion of four amino acids is at amino acid position 1181 in the outermost loops of the NDs that make up the pore-forming loops at the tip of the TcA (Figure 1B). According to current understanding, this region of the TcA contacts the target receptor before the membrane perforation [12,14]. After verifying a near identical size exclusion chromatography (SEC) peak in comparison to Xn-XptA2 wt (Figure 1C), we attempted to measure the binding affinity of the Xn-XptA2 YWKT chimera (hereafter denoted as Xn-XptA2 YWKT) to rat pancreatic AR42J cells expressing SSTR2. We measured the binding affinity of this construct to AR42J cells by using a Lindmo assay after conjugating Xn-XptA2 YWKT to deferoxamine (DFO) and radiolabeling with Zirconium-89 (^89^Zr) [26,27,28,37] (Figure 1D) (Figure S1A). The conjugation of DFO to Xn-XptA2 YWKT was performed by combining an 8-fold molar excess of chelator at pH 9 for 1 h at 37 °C. Subsequent labeling with ^89^Zr yielded a maximum specific activity of 74 MBq/mg (2 mCi/mg) with 100% radiochemical yield via iTLC (Figure S1B). The immunoreactive fraction was observed to be 2.8%, indicating no specific binding of Xn-XptA2 YWKT to SSTR2. A reduction of the amount of DFO used to a 2-fold molar excess still yielded a 100% radiochemical yield at a 74 MBq/mg (2 mCi/mg) specific activity but did not change the results of the Lindmo assay (Figure S1C,D). The lack of binding to the SSTR2+ AR42J cells suggests the binding affinity of Xn-XptA2 YWKT is easily disrupted, leading us to question the structural stability of the chimeric TcA.

**Figure 2:**
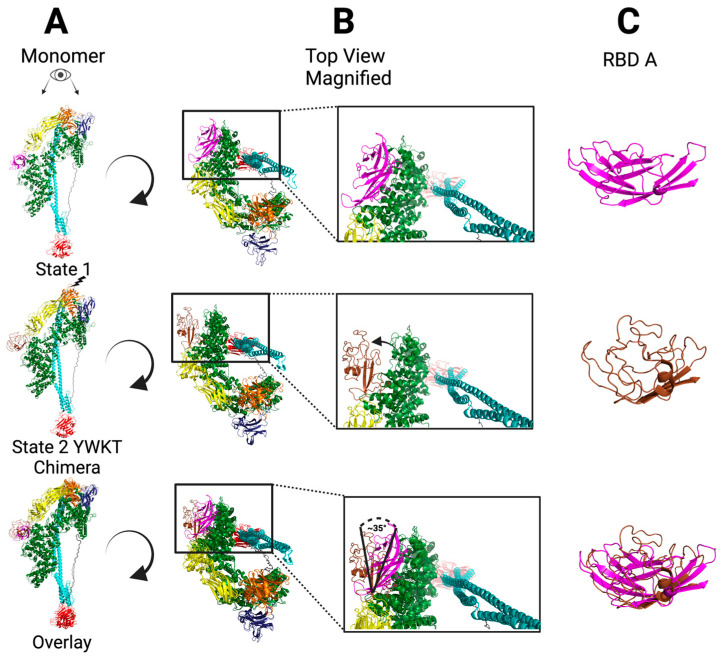
Structural representation of the RBD A flip in the YWKT chimeric structure. (**A**) (Top to Bottom) Side view of the Xn-XptA2 State 1 (wt) monomer (8TQE) colored by domain, side view of the Xn-XptA2 YWKT State 2 monomer colored by domain, Side view of the Xn-XptA2 State 1 (wt) monomer (8TQE) colored by domain overlaid with the side view of the Xn-XptA2 YWKT State 2 monomer colored by domain. (**B**) (Top to Bottom) Top view of the Xn-XptA2 State 1 (wt) monomer (8TQE) colored by domain zoomed in on RBD A (magenta), Top view of the Xn-XptA2 YWKT State 2 monomer colored by domain zoomed in on RBD A (brown), Top view of the Xn-XptA2 State 1 (wt) monomer (8TQE) colored by domain overlaid with the top view of the Xn-XptA2 YWKT State 2 monomer colored by domain zoomed in on RBD A displaying a ~45° flip between the domains. (**C**) (Top to Bottom) RBD A of Xn-XptA2 State 1, RBD A of Xn-XptA2 YWKT State 2, overlay of RBD A between the State 1 and State 2 structures displaying the structural rearrangement within the domain.

To test whether the structural integrity of Xn-XptA2 YWKT was intact, we solved the structure to 4.3 Å resolution using cryo-EM single particle analysis (SPA). We observe a mostly homogeneous structure between Xn-XptA2 wt (8TQE) and Xn-XptA2 YWKT with ~6% difference in amino acid structural composition overall through RMSD analysis. The biggest deviation in structure lies between G298 and P440. This stretch of 142 amino acids comprises RBD A on the periphery of the outer shell. We observe a major structural rearrangement of RBD A as well as the full domain pivoting ~35° out of the shell compared to Xn-XptA2 wt (8TQE) (Figure 2) [12]. The structural rearrangement of RBD A in Xn-XptA2 YWKT consists mainly of a significant loss in secondary structure in the form of β-sheets. In the Xn-XptA2 wt RBD A (G298-P440), β-sheets are observed at positions I315-V320, L327-T337, N345-Y351, Q356-A361, G370-L373, V383-L386, K414-Y420, and A426-P440. In contrast, β-sheets in the Xn-XptA2 YWKT RBD A are only observed at positions G328-V335 and I433-P440 with the former regions of β-sheets rearranging into loops (Figure S2). The single region containing an α-helix in the Xn-XptA2 wt structure (S407-Y409) is conserved in the Xn-XptA2 YWKT structure (S405-G412). RBD A is ~130 angstroms and 742 residues away from the outer loops of the ND at the YWKT insertion site (P440-T1181), indicating that disruption of these outer loops has an allosteric effect on upstream domains. Previous work suggests that the NDs could serve as an electrostatic lock mediated by electrostatic interactions between the NDs on each protomer [36]. To make the Xn-XptA2 YWKT construct we combined a strategy of inserting the pharmacophore sequence into the desired position and additionally mutating one native residue in order to modify the amino acid composition of the outer loops of the NDs. We inserted the YWKT amino acids in between Threonine at position 1181 (T1181) and Proline at position 1183 (P1183) with the wt Aspartic Acid at position 1182 (D1182) replaced by the Threonine (T) in the YWKT sequence. Although this strategy alters the electrostatic composition of the outer loops of the NDs significantly, we did not observe any repulsion of the NDs between protomers.

**Figure 3:**
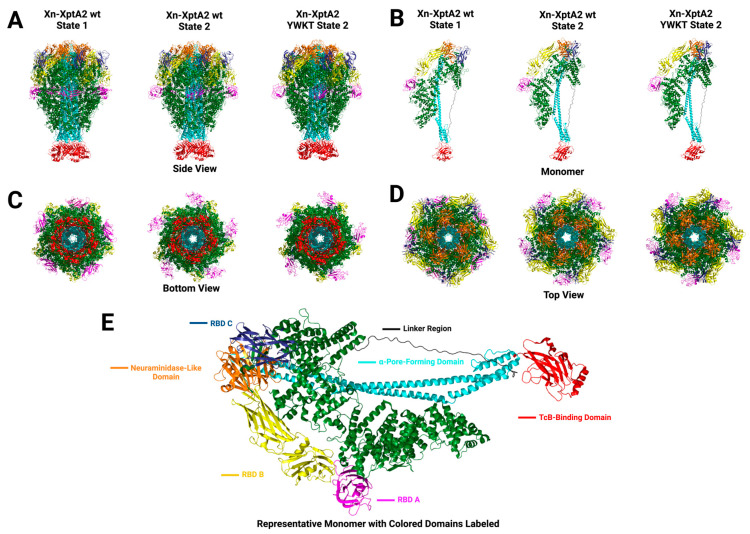
Structural comparisons of Xn-XptA2 wt and Xn-XptA2 YWKT in State 1 and State 2. (**A**) Side view of Xn-XptA2 wt (State 1 and State 2) and Xn-XptA2 YWKT (State 2) colored by domain. (**B**) Monomer representation of each Xn-XptA2 TcA colored by domain. (**C**) Bottom view of Xn-XptA2 structures colored by domain. (**D**) Top view of Xn-XptA2 structures colored by domain. (**E**) Representative Xn-XptA2 wt State 2 monomer colored by domain with each domain labeled.

To ascertain whether this intermediate state is physiological, we analyzed the original Xn-XptA2 published wt cryo-EM data set to determine if the alternative State 2 is present in the data (8TQE) [12]. We separated particle subsets based on structural variations in 2D and 3D classifications indicative of RBD A mobility and observed the State 2 intermediate in the Xn-XptA2 wt data. We were able to resolve the Xn-XptA2 wt State 2 structure to 3.9 Å resolution, leading us to conclude that the State 2 structure is a physiological intermediate state preceding the pore state conformational change in Xn-XptA2 wt (Figure 3). We observe an almost identical ~35° pivot at RBD A as well as a major structural rearrangement that is unique from the Xn-XptA2 YWKT State 2 structure and aligns more with the Xn-XptA2 wt State 1 RBD A. Comparing the Xn-XptA2 wt State 2 structural rearrangement of RBD A to the Xn-XptA2 wt State 1 RBD A, we observe that all of the eight β-sheets and the single α-helix found in the Xn-XptA2 wt State 1 RBD A are maintained, with the exception of the last two β-sheets being separated by a loop that leads to nine total separate β-sheets within RBD A (Figure S2).

**Figure 4:**
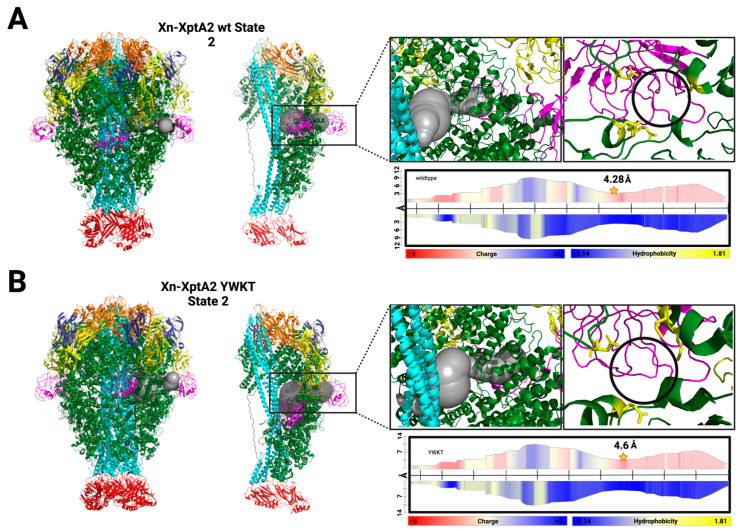
Analysis of the pore opening after RBD A conformational change. (**A**) (Left to Right) Xn-XptA2 wt State 2 pentamer colored by domain displaying one tunnel through Chain A. Two monomers displaying the tunnel formation showing zoomed-in views of the RBD A opening with and without the tunnel present. Residues making up the narrowest constriction site are colored yellow and displayed as sticks. Pore diameter representation output from MOLE colored by charge and hydrophobicity highlighting the 4.28 Å constriction site radius within the pore. (**B**) (Left to Right) Xn-XptA2 YWKT State 2 pentamer colored by domain displaying one tunnel through Chain A. Two monomers displaying the tunnel formation showing zoomed-in views of the RBD A opening with and without the tunnel present. Residues making up the narrowest constriction site are colored yellow and displayed as sticks. Pore diameter representation output from MOLE colored by charge and hydrophobicity highlighting the 4.6 Å constriction site radius within the pore.

Upon closer inspection of the region around RBD A in both Xn-XptA2 State 2 structures, we observed an opening in the outer shell of the protein where RBD A pivots outward (Figure 4). The narrowest area of this pore in the Xn-XptA2 wt State 2 structure has a maximum radius of 4.28 Å that is surrounded by four amino acids from two separate protomers: D518 (Chain A), Q152 (Chain E), D156 (Chain E), and S291 (Chain A) (Figure 4A). Comparatively, in the Xn-XptA2 YWKT structure, the narrowest constriction point in the pore has a maximum radius of 4.6 Å and is also compressed by four amino acids from two separate protomers: D518 (Chain A), Q152 (Chain E), Q294 (Chain A), and K291 (Chain A) (Figure 4B). This pore leads into the inner cavity space between the outer shell and the outside of the α-pore forming domains that make up the translocation channel. This opening would allow for the outer solvent to enter the inner cavity containing the linker region that would otherwise be shielded from outside solvent.

### 3.2. Analyses of Receptor Binding Domain Substitution into Xn-XptA2

**Figure 5:**
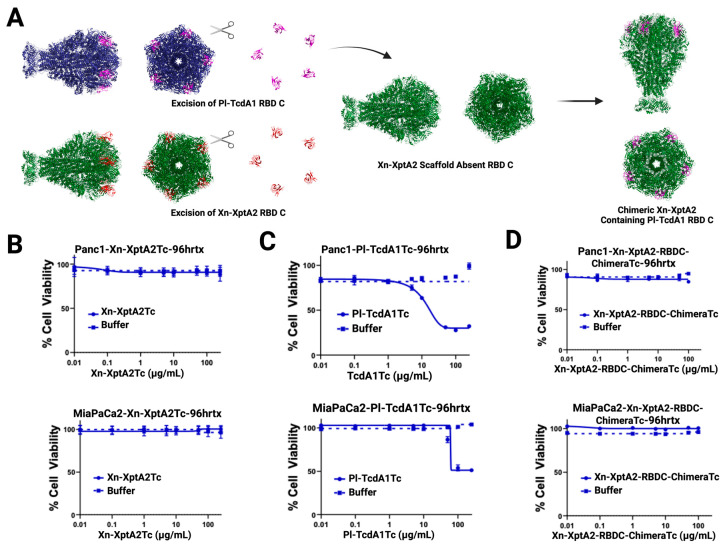
Overview of RBD C chimera formation and pancreatic cancer cell viability assays. (**A**) Summarization of the strategy used to modify Xn-XptA2 wt to express Pl-TcdA1 RBD C using high-resolution structures. (**B**–**D**) Sensitivity of Panc1 and MiaPaCa2 cells to Xn-XptA2Tc (**B**), PI-TcdA1Tc (**C**), and Xn-XptA2-RBDC-ChimeraTc (**D**). Cell viability was assessed by alamarBlue assays after 96 hr exposure. N = 3 independent experiments (**B**–**D**).

The second strategy we implemented to alter the host tropism of Xn-XptA2 was to substitute a full RBD from the homologous TcA *Photorhabdus luminescens* TcdA1 (Pl-TcdA1) into Xn-XptA2. Pl-TcdA1 is unambiguously the best-characterized TcA in the field to date and shares 43.66% amino acid sequence identity with Xn-XptA2 [14,15,21,36,38]. Furthermore, TcdA1 displays poly-specificity to various cell lines, while Xn-XptA2 seems to have little variation in targeting specificity, making the determination of altered host tropism seemingly straightforward [11,39,40,41]. Regarding the domain substitution, we decided to substitute the Xn-XptA2 RBD C (F1659-G1787) for the analogous RBD D (A1643-N1752) in Pl-TcdA1 due to these RBDs being closest to the membrane before pore formation [36] (Figure 5 and Figure S3). This was achieved by excising the sequence of DNA encoding the TcdA1 RBD D and ligating this sequence into the scaffold sequence encoding for Xn-XptA2 after excision of the homologous RBD C sequence. After sequence verification, the Xn-XptA2 RBD chimera was expressed and purified using the same method as with Xn-XptA2 wt. High-resolution structure determination of the Xn-XptA2 RBD C chimera was achieved through cryo-EM single particle analysis, yielding a final high-resolution structure at 3.6 Å resolution (Figure 5). Structural analysis displayed a stable chimera in the State 1 conformation, indicating that the full RBD substitution did not have an allosteric effect on alternative domains. Upon further inspection, the Pl-TcdA1 RBD D within the Xn-XptA2 scaffold was mostly identical in structure to Pl-TcdA1 wt RBD D, with all β-sheets being conserved and the loss of one α-helix at position N1651-G1656 (Figure S3).

After confirming the structural integrity of the Xn-XptA2 RBD C chimera, all three TcAs (Xn-XptA2 wt, Pl-TcdA1 wt, and Xn-XptA2 RBD C chimera) were mixed with purified Pl-TcdB2-TccC3 (TcB-TcC) at 5:1 TcA:TcB-TcC ratios to form full ABC toxin complexes. Sheets et al. showed that Xn-XptA2 could form a functional hybrid Tc with Pl-TcdB2-TccC3 based on toxicity studies with *Helicoverpa zea* larvae [11]. After verification of Tc formation for all three TcAs through SEC, the Tcs were used for cell viability assays on Panc1 and MiaPaCa2 cells for 96 h at concentrations ranging from 0 to 100 μg/mL (Figure 5). As expected, Xn-XptA2 Tc had no effect on cell viability for either cell line, while Pl-TcdA1 Tc displayed toxicity to both Panc1 and MiaPaCa2 cells at ~30 μg/mL and ~70 μg/mL, respectively. When the Panc1 and MiaPaCa2 cells were exposed to the Xn-XptA2 RBD C chimera Tc, we observed no significant difference in cell viability for both cell lines. Due to the structural integrity of Pl-TcdA1 RBD D within the Xn-XptA2 RBD C chimera, we conclude that RBD C is not responsible for host tropism to Panc1 and MiaPaCa2 cells through the process of elimination. Although alternative host tropism was not observed, we show that RBD substitutions between TcAs can serve as viable options for understanding which RBDs are necessary to target cell surface receptors. Furthermore, we display the capability to substitute alternative RBDs into the Xn-XptA2 scaffold while maintaining structural stability.

### 3.3. Examination of Xn-XptA2 in Mice

The unique mechanism of ABC Tcs makes these proteins ideal candidates for targeted peptide delivery. Although the structural and mechanistic studies of ABC Tcs have progressed rapidly over the past decade, our current understanding of these Tcs is still at an early stage regarding how these proteins affect mammalian organisms. Previous studies show that the targeting specificity of ABC Tcs varies significantly between homologs, with some Tcs, such as Pl-TcdA1, displaying a broad targeting range and others, such as Xn-XptA2, displaying a much narrower range of specificity [11,14,39,40].

Studies regarding Xn-XptA2 have focused on the insecticidal toxicity of the Tc, displaying a limited range of specificity to *Helicoverpa zea* (corn earworm) and *Heliothis viriscens* (tobacco budworm), indicating that there is a specific mechanism of host tropism [11]. The narrow range of host tropism in Xn-XptA2 is well-suited for clinical application because of its specific targeting combined with the broad cargo-carrying and translocation capabilities of the Tc.

**Figure 6:**
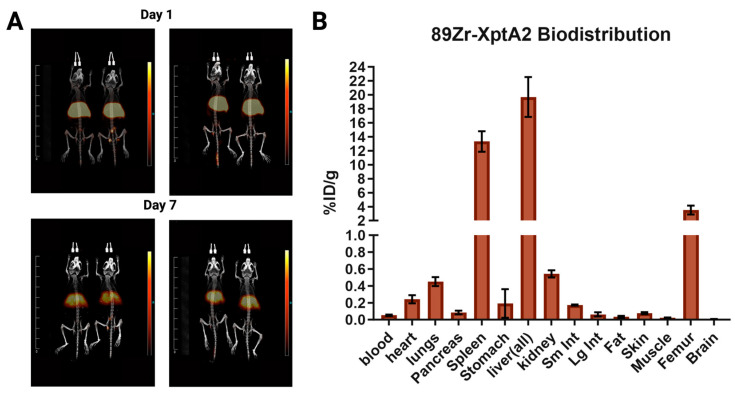
PET imaging and biodistribution of Zirconium-89 radiolabeled Xn-XptA2 in mice. (**A**) Small animal positron emission tomography image of Balb/C mice after tail vein injection with Zirconium-89 radiolabeled Xn-XptA2 displaying representative images at day 1 and day 7 (*n* = 4). (**B**) Biodistribution displaying radioactivity remaining in each organ after day 7.

To achieve a basic understanding of the effects of Xn-XptA2 in mammals, we conjugated Xn-XptA2 to DFO and radiolabeled the protein with ^89^Zr. After radiolabeling, the protein was injected into mice (*n* = 4) via injection into the tail vein, and animals were imaged with positron emission tomography (PET) over a seven-day period (Figure 6A). After day seven, animals were euthanized, and their organs were scanned for radioactivity to identify the location of the ^89^Zr Xn-XptA2 protein (Figure 6B) [26,27,28]. Imaging and biodistribution results suggest hepatic clearance, with a large portion of the activity residing in the liver (19.7% ID/g). Spleen uptake was also observed but was no higher than other ^89^Zr-labeled antibodies reported in the literature [25,42,43]. Low accumulation was observed in other organs 7 days post-injection. Based on these data, we conclude that Xn-XptA2 wt was well-tolerated by the mice, has a similar uptake pattern as antibody conjugates, and most likely undergoes hepatic clearance as is common for large proteins.

## 4. Discussion

ABC toxin complexes display a unique tri-modal mechanism that allows them to target cell surface receptors, shuttle peptides while shielding them from environmental stressors, and translocate these peptides across a membrane bilayer [1,14,15,38,44,45]. Most biologics used for the purpose of therapeutic applications are either unimodal, such as antibodies that target cell surface receptors for immune cell recognition, or bimodal such as peptide conjugates that contain a cytotoxic reagent meant to eliminate target cells after recognition of the cell surface [46,47,48]. The pitfalls with using these approaches usually consist of premature degradation and clearance, nonspecific/off-target effects, or lack of a rapid cytotoxic response from immune cell activation [48,49,50,51]. Although ABC Tcs have been extensively studied over the past few decades, additional work is necessary to determine if these proteins can be used effectively in a therapeutic setting. The exact targeting mechanism and cell surface receptors for most ABC Tcs are unclear, and host tropism varies between closely related homologs, further complicating our understanding of targeting across various Tcs. In this manuscript, we outline two separate strategies to modify the targeting capabilities of Xn-XptA2, a highly specific TcA known to target only the tobacco budworm and the corn earworm.

The first strategy consists of mutating the outer loops of the neuraminidase domain on each chain of the TcA to express the SSTR2 targeting pharmacophore sequence of TATE. Although our attempts to bind this construct to SSTR2+ AR42J cells were unsuccessful, we were able to glean important information regarding the conformational dynamics and structural stability of Xn-XptA2. We found that perturbing the NDs with this mutation causes Xn-XptA2 to be trapped in an alternative conformation that has never been observed for this protein. Furthermore, this alternative state “State 2” was observed in the Xn-XptA2 wt dataset, showing that this conformation is indeed a physiological alternative state in the data for wt Xn-XptA2. The site at which conformational change occurs is far upstream in the amino acid sequence from the site at which the neuraminidase domains were perturbed in the Xn-XptA2 YWKT mutant, suggesting that perturbing the NDs has a specific allosteric effect on RBD A.

The NDs are located at the tip of the TcA and based on our current understanding of membrane perforation of TcAs, would most likely encounter cell surface receptors or the membrane of the target cell [12,14]. This makes it reasonable to conclude that once Xn-XptA2 contacts cell surface receptors or the cell membrane, this mechanical stress stimulates Xn-XptA2 to undergo a conformational change from the canonical pre-pore state or State 1 to the newly discovered alternative State 2. Furthermore, once this intermediate state is achieved, an opening is observed between the outer shell of Xn-XptA2 and the inner α-pore forming domains that make up the translocation channel. Within this area of the TcA resides the linker region, which is suggested to be the driving force for conformational change [21]. This suggests that initial mechanical stress on the NDs through contact with other proteins or biomolecules triggers RBD A to re-orient to allow for solvent from the environment to enter the inner cavity containing the linker region. From this, we propose that once Xn-XptA2 encounters a target receptor, the protein undergoes a conformational change from State 1 to State 2 to allow an influx of solvent, presumably in the insect midgut at very basic pH levels, into the inner cavity to further stimulate an additional conformational change leading into the pore state transition. From a bio-engineering perspective, the conformational change could be problematic as we observe a significant structural rearrangement of RBD A within the Xn-XptA2 YWKT mutant that is not present in the Xn-XptA2 wt structures (Figure S2). Alternatively, if RBD A is an integral RBD necessary for host tropism, then the State 2 conformation may serve as a physiological mechanism to sample space from the outside environment. If RBD A is an integral RBD for host recognition, then structural rearrangement serves as a major barrier to altering host tropism. Due to the observed dynamic changes in the Xn-XptA2 structure when the outer scaffold of the TcA is genetically altered, an alternative strategy involving the fusion of a cancer-targeting peptide could be advantageous in altering host tropism. Further studies would be necessary to identify the effects and efficacy of this approach in relation to the structural integrity and translocation capabilities of a peptide fused Xn-XptA2.

The second strategy we implemented to alter host tropism is like the strategy that Song et al. utilized to determine that RBD D from Pl-TcdA1 (W14) is responsible for host tropism to HeLa cells through interactions with *N*-glycan [39]. Coupling this knowledge with the fact that Pl-TcdA1 RBD D is the RBD that is most proximal to the membrane prior to membrane perforation, we decided to substitute the homologous Xn-XptA2 RBD C for the Pl-TcdA1 RBD D. Our 3.6 Å resolution structure revealed that not only was the RBD substitution successful but this substitution was achieved while maintaining the structural integrity of the chimeric TcA in the State 1 conformation. After confirming the structural integrity of the modified Pl-TcdA1 RBD D in the Xn-XptA2 scaffold, we performed cell viability experiments on pancreatic cancer cells (Panc1 and MiaPaCa2) using all three TcAs in complex with Pl-TcdB2-TccC3. We confirmed that the Xn-XptA2 hybrid Tc did not have any cytotoxic effect on the cells, whereas Pl-TcdA1 Tc displayed toxicity at ~30–70 μg/mL. Unexpectedly, the results from the pancreatic cancer cell viability assays after exposure to the Xn-XptA2 RBD C chimera Tc containing the Pl-TcdA1 RBD D did not have any effect on cell viability. This suggests that the toxicity observed on both cell lines after exposure to Pl-TcdA1 Tc is not mediated by RBD-D, indicating multiple methods of host tropism for varying cell lines. Nevertheless, we demonstrate that Xn-XptA2 can be used as a protein scaffold for substituting in alternative RBDs or IgG-like domains while maintaining structural stability.

Finally, we investigated the biodistribution of radiolabeled Xn-XptA2 wt in mice. The biodistribution obtained after day 7 displayed a predictable pattern of hepatic clearance that is common for large proteins as well as an organ accumulation profile that is common to antibody conjugates studied for therapeutic applications [25,42,43]. To our knowledge, this is the first study analyzing the effects and localization of any TcA in a mammal. These data set the stage for the future use of mice models, once alternative host tropism is achieved in TcAs.

ABC Tcs are unique candidates for protein engineering that could potentially lead to translational applications. Xn-XptA2 is a clear contender from this class of toxins because of its efficacy with only two model insect species. This specificity will presumably decrease off-target effects. Here, we describe two potential pathways for altering the host tropism of Xn-XptA2 and show unique structural characteristics of this TcA that have not been observed in any other TcA. These data lead to a better understanding of the dynamic mechanism of TcAs and, thus, a better understanding of how to utilize TcAs in a clinical or biotechnological setting. Our data show that RBD substitutions are possible across TcA homologs while maintaining structural stability. We also demonstrate that Xn-XptA2 can be used in vivo in mice, a discovery that sets the stage for future use in a clinical context.

## Data Availability

The raw data supporting the conclusions of this article will be made available by the authors upon request.

## Supplementary Materials

The following supporting information can be downloaded at: https://www.mdpi.com/article/10.3390/biotech14010005/s1, Figure S1: Overview of Xn-XptA2 and Xn-XptA2 YWKT Radiolabeling and Lindmo Assay; Figure S2: Comparison of Secondary Structure in Xn-XptA2 RBD A; Figure S3: Xn-XptA2 RBD C Chimera Overview; Table S1: Cryo-EM Data Collection and Refinement Statistics for Wild-Type Xn-XptA2; Table S2: Cryo-EM Data Collection and Refinement Statistics for both Xn-XptA2 YWKT and Xn-XptA2 RBD C Chimera; Figure S4: Cryo-EM Processing and Analysis of Xn-XptA2 RBD C Chimera; Figure S5: Cryo-EM Processing and Analysis of Xn-XptA2 YWKT Chimera; Figure S6: Cryo-EM Processing and Analysis of Xn-XptA2 wt State 2; Figure S7: Cryo-EM Processing and Analysis of Xn-XptA2 State 2 wt.

## References

[1] Leidreiter F., Roderer D., Meusch D., Gatsogiannis C., Benz R., Raunser S. (2019). Common architecture of Tc toxins from human and insect pathogenic bacteria. Sci. Adv..

[2] Ffrench-Constant R., Waterfield N., Laskin A.I., Bennett J.W., Gadd G.M., Sariaslani S. (2005). An ABC Guide to the Bacterial Toxin Complexes. Advances in Applied Microbiology.

[3] Bowen D., Rocheleau T.A., Blackburn M., Andreev O., Golubeva E., Bhartia R., Ffrench-Constant R.H. (1998). Insecticidal toxins from the bacterium *Photorhabdus luminescens*. Science.

[4] Rangel L.I., Henkels M.D., Shaffer B.T., Walker F.L., Davis E.W., Stockwell V.O., Bruck D., Taylor B.J., Loper J.E. (2016). Characterization of Toxin Complex Gene Clusters and Insect Toxicity of Bacteria Representing Four Subgroups of Pseudomonas fluorescens. PLoS ONE.

[5] Waterfield N., Hares M., Hinchliffe S., Wren B., Ffrench-Constant R. (2007). The insect toxin complex of Yersinia. Adv. Exp. Med. Biol..

[6] Ffrench-Constant R.H., Waterfield N., Burland V., Perna N.T., Daborn P.J., Bowen D., Blattner F.R. (2000). A genomic sample sequence of the entomopathogenic bacterium *Photorhabdus luminescens* W14: Potential implications for virulence. Appl. Environ. Microbiol..

[7] Forst S., Dowds B., Boemare N., Stackebrandt E. (1997). *Xenorhabdus* and *Photorhabdus* spp.: Bugs that kill bugs. Annu. Rev. Microbiol..

[8] Joyce S.A., Watson R.J., Clarke D.J. (2006). The regulation of pathogenicity and mutualism in *Photorhabdus*. Curr. Opin. Microbiol..

[9] Morgan J.A., Sergeant M., Ellis D., Ousley M., Jarrett P. (2001). Sequence analysis of insecticidal genes from Xenorhabdus nematophilus PMFI296. Appl. Environ. Microbiol..

[10] Sergeant M., Jarrett P., Ousley M., Morgan J.A. (2003). Interactions of insecticidal toxin gene products from Xenorhabdus nematophilus PMFI296. Appl. Environ. Microbiol..

[11] Sheets J.J., Hey T.D., Fencil K.J., Burton S.L., Ni W., Lang A.E., Benz R., Aktories K. (2011). Insecticidal toxin complex proteins from Xenorhabdus nematophilus: Structure and pore formation. J. Biol. Chem..

[12] Martin C.L., Chester D.W., Radka C.D., Pan L., Yang Z., Hart R.C., Binshtein E.M., Wang Z., Nagy L., DeLucas L.J. (2023). Structures of the Insecticidal Toxin Complex Subunit XptA2 Highlight Roles for Flexible Domains. Int. J. Mol. Sci..

[13] Piper S.J., Brillault L., Rothnagel R., Croll T.I., Box J.K., Chassagnon I., Scherer S., Goldie K.N., Jones S.A., Schepers F. (2019). Cryo-EM structures of the pore-forming A subunit from the Yersinia entomophaga ABC toxin. Nat. Commun..

[14] Gatsogiannis C., Merino F., Prumbaum D., Roderer D., Leidreiter F., Meusch D., Raunser S. (2016). Membrane insertion of a Tc toxin in near-atomic detail. Nat. Struct. Mol. Biol..

[15] Martin C.L., Hill J.H., Aller S.G. (2024). Host Tropism and Structural Biology of ABC Toxin Complexes. Toxins.

[16] Gatsogiannis C., Merino F., Roderer D., Balchin D., Schubert E., Kuhlee A., Hayer-Hartl M., Raunser S. (2018). Tc toxin activation requires unfolding and refolding of a β-propeller. Nature.

[17] Busby J.N., Panjikar S., Landsberg M.J., Hurst M.R.H., Lott J.S. (2013). The BC component of ABC toxins is an RHS-repeat-containing protein encapsulation device. Nature.

[18] Zahaf N.-I., Lang A.E., Kaiser L., Fichter C.D., Laßmann S., McCluskey A., Augspach A., Aktories K., Schmidt G. (2017). Targeted delivery of an ADP-ribosylating bacterial toxin into cancer cells. Sci. Rep..

[19] Roderer D., Hofnagel O., Benz R., Raunser S. (2019). Structure of a Tc holotoxin pore provides insights into the translocation mechanism. Proc. Natl. Acad. Sci. USA.

[20] Roderer D., Raunser S. (2019). Tc Toxin Complexes: Assembly, Membrane Permeation, and Protein Translocation. Annu. Rev. Microbiol..

[21] Gatsogiannis C., Lang A.E., Meusch D., Pfaumann V., Hofnagel O., Benz R., Aktories K., Raunser S. (2013). A syringe-like injection mechanism in *Photorhabdus luminescens* toxins. Nature.

[22] Roderer D., Schubert E., Sitsel O., Raunser S. (2019). Towards the application of Tc toxins as a universal protein translocation system. Nat. Commun..

[23] Ng’ang’a P.N., Ebner J.K., Plessner M., Aktories K., Schmidt G. (2019). Engineering *Photorhabdus luminescens* toxin complex (PTC) into a recombinant injection nanomachine. Life Sci. Alliance.

[24] Marquez B.V., Ikotun O.F., Zheleznyak A., Wright B., Hari-Raj A., Pierce R.A., Lapi S.E. (2014). Evaluation of (89)Zr-pertuzumab in Breast cancer xenografts. Mol. Pharm..

[25] Chang A.J., Desilva R., Jain S., Lears K., Rogers B., Lapi S. (2012). 89Zr-Radiolabeled Trastuzumab Imaging in Orthotopic and Metastatic Breast Tumors. Pharmaceuticals.

[26] Ducharme M., Hall L., Eckenroad W., Cingoranelli S.J., Houson H.A., Jaskowski L., Hunter C., Larimer B.M., Lapi S.E. (2023). Evaluation of [(89)Zr]Zr-DFO-2Rs15d Nanobody for Imaging of HER2-Positive Breast Cancer. Mol. Pharm..

[27] Gimblet G.R., Houson H.A., Whitt J., Reddy P., Copland J.A., Kenderian S.S., Szkudlinski M.W., Jaskula-Sztul R., Lapi S.E. (2024). PET Imaging of Differentiated Thyroid Cancer with TSHR-Targeted [(89)Zr]Zr-TR1402. Mol. Pharm..

[28] Massicano A.V.F., Song P.N., Mansur A., White S.L., Sorace A.G., Lapi S.E. (2023). [(89)Zr]-Atezolizumab-PET Imaging Reveals Longitudinal Alterations in PDL1 during Therapy in TNBC Preclinical Models. Cancers.

[29] Queern S.L., Aweda T.A., Massicano A.V.F., Clanton N.A., El Sayed R., Sader J.A., Zyuzin A., Lapi S.E. (2017). Production of Zr-89 using sputtered yttrium coin targets. Nucl. Med. Biol..

[30] Miller A.L., Fehling S.C., Garcia P.L., Gamblin T.L., Council L.N., van Waardenburg R., Yang E.S., Bradner J.E., Yoon K.J. (2019). The BET inhibitor JQ1 attenuates double-strand break repair and sensitizes models of pancreatic ductal adenocarcinoma to PARP inhibitors. EBioMedicine.

[31] Miller A.L., Fehling S.C., Vance R.B., Chen D., Brown E.J., Hossain M.I., Heard E.O., Andrabi S.A., Wang H., Yang E.S. (2024). BET inhibition decreases HMGCS2 and sensitizes resistant pancreatic tumors to gemcitabine. Cancer Lett..

[32] Zhang L., Vines D.C., Scollard D.A., McKee T., Komal T., Ganguly M., Do T., Wu B., Alexander N., Vali R. (2017). Correlation of Somatostatin Receptor-2 Expression with Gallium-68-DOTA-TATE Uptake in Neuroblastoma Xenograft Models. Contrast Media Mol. Imaging.

[33] Reubi J.C., Schär J.C., Waser B., Wenger S., Heppeler A., Schmitt J.S., Mäcke H.R. (2000). Affinity profiles for human somatostatin receptor subtypes SST1-SST5 of somatostatin radiotracers selected for scintigraphic and radiotherapeutic use. Eur. J. Nucl. Med..

[34] Maecke H.R., Reubi J.C. (2011). Somatostatin receptors as targets for nuclear medicine imaging and radionuclide treatment. J. Nucl. Med..

[35] Torre B.G., Albericio F. (2021). The Pharmaceutical Industry in 2020. An Analysis of FDA Drug Approvals from the Perspective of Molecules. Molecules.

[36] Meusch D., Gatsogiannis C., Efremov R.G., Lang A.E., Hofnagel O., Vetter I.R., Aktories K., Raunser S. (2014). Mechanism of Tc toxin action revealed in molecular detail. Nature.

[37] Lindmo T., Boven E., Cuttitta F., Fedorko J., Bunn P.A. (1984). Determination of the immunoreactive fraction of radiolabeled monoclonal antibodies by linear extrapolation to binding at infinite antigen excess. J. Immunol. Methods.

[38] Lang A.E., Konukiewitz J., Aktories K., Benz R. (2013). TcdA1 of *Photorhabdus luminescens*: Electrophysiological analysis of pore formation and effector binding. Biophys. J..

[39] Song N., Chen L., Ren X., Waterfield N.R., Yang J., Yang G. (2021). N-Glycans and sulfated glycosaminoglycans contribute to the action of diverse Tc toxins on mammalian cells. PLoS Pathog..

[40] Roderer D., Bröcker F., Sitsel O., Kaplonek P., Leidreiter F., Seeberger P.H., Raunser S. (2020). Glycan-dependent cell adhesion mechanism of Tc toxins. Nat. Commun..

[41] Ng’ang’a P.N., Siukstaite L., Lang A.E., Bakker H., Römer W., Aktories K., Schmidt G. (2021). Involvement of N-glycans in binding of *Photorhabdus luminescens* Tc toxin. Cell Microbiol..

[42] Ghosh S., Fletcher N.L., Huda P., Houston Z.H., Howard C.B., Lund M.E., Lu Y., Campbell D.H., Walsh B.J., Thurecht K.J. (2023). Pharmacokinetics and Biodistribution of (89)Zr-Miltuximab and Its Antibody Fragments as Glypican-1 Targeting Immuno-PET Agents in Glioblastoma. Mol. Pharm..

[43] Ghai A., Maji D., Cho N., Chanswangphuwana C., Rettig M., Shen D., DiPersio J., Akers W., Dehdashti F., Achilefu S. (2018). Preclinical Development of CD38-Targeted [(89)Zr]Zr-DFO-Daratumumab for Imaging Multiple Myeloma. J. Nucl. Med..

[44] Aleksandrova N.A., Roche S.G., Low Y.S., Landsberg M.J. (2023). Recent insights into mechanisms of cellular toxicity and cell recognition associated with the ABC family of pore-forming toxins. Biochem. Soc. Trans..

[45] Chassagnon I.R., Piper S.J., Landsberg M.J., Andrews D.L., Lipson R.H., Nann T. (2019). 2.13—ABC Toxins: Self-Assembling Nanomachines for the Targeted Cellular Delivery of Bioactive Proteins. Comprehensive Nanoscience and Nanotechnology.

[46] Monroe M.K., Wang H., Anderson C.F., Jia H., Flexner C., Cui H. (2022). Leveraging the therapeutic, biological, and self-assembling potential of peptides for the treatment of viral infections. J. Control Release.

[47] Gattu R., Ramesh S.S., Nadigar S., Ramesh S. (2023). Conjugation as a Tool in Therapeutics: Role of Amino Acids/Peptides-Bioactive (Including Heterocycles) Hybrid Molecules in Treating Infectious Diseases. Antibiotics.

[48] Chen Z., Kankala R.K., Yang Z., Li W., Xie S., Li H., Chen A.Z., Zou L. (2022). Antibody-based drug delivery systems for cancer therapy: Mechanisms, challenges, and prospects. Theranostics.

[49] Zeller T., Lutz S., Münnich I.A., Windisch R., Hilger P., Herold T., Tahiri N., Banck J.C., Weigert O., Moosmann A. (2022). Dual checkpoint blockade of CD47 and LILRB1 enhances CD20 antibody-dependent phagocytosis of lymphoma cells by macrophages. Front. Immunol..

[50] Wu C., Wang M., Sun J., Jia Y., Zhu X., Liu G., Zhu Y., Guan Y., Zhang Z., Pang X. (2023). Peptide-drug co-assembling: A potent armament against cancer. Theranostics.

[51] Wang L., Wang N., Zhang W., Cheng X., Yan Z., Shao G., Wang X., Wang R., Fu C. (2022). Therapeutic peptides: Current applications and future directions. Signal Transduct. Target. Ther..

