# Supplementary material for: Evaluating TcAs for Use in Biotechnology Applications
Source: BioTech (Basel). 2025 Jan 25;14(1):5. doi: 10.3390/biotech14010005 (PMC11843870; doi:10.3390/biotech14010005)
Supplement: Supplementary file 1 [file biotech-14-00005-s001.zip › biotech-3398843-supplementary.pdf]

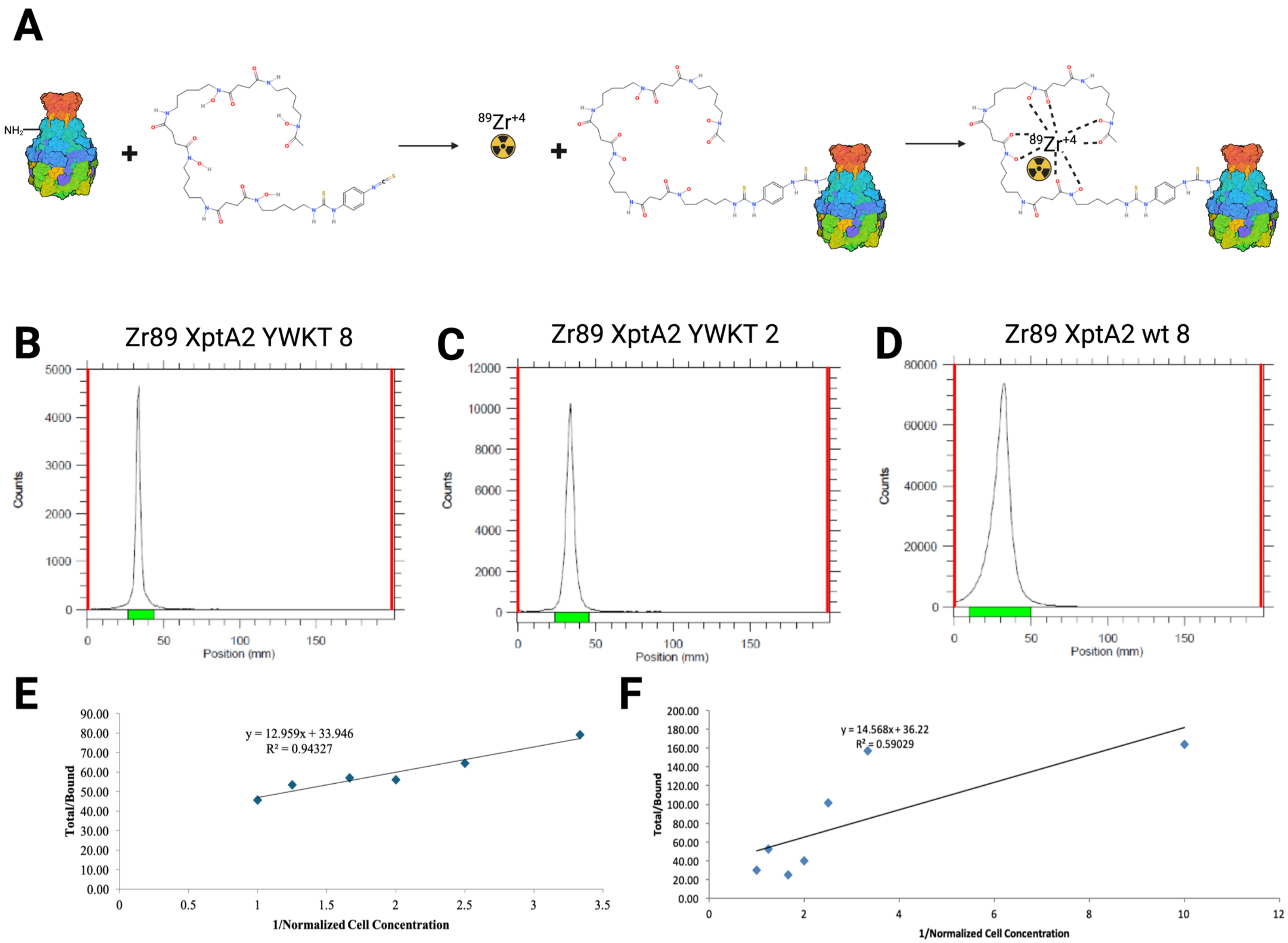

Figure S1. Overview of Xn-XptA2 and Xn-XptA2 YWKT Radiolabeling and Lindmo Assay. A. Schematic overview of DFO conjugation and radiolabeling Xn-XptA2 with Zr89. B. iTLC displaying 100% radiochemical yield of Xn-XptA2 YWKT using 8-fold molar excess of DFO. C. iTLC displaying 100% radiochemical yield of Xn-XptA2 YWKT using 2-fold molar excess of DFO. D. iTLC displaying 100% radiochemical yield of Xn-XptA2 wt using 8-fold molar excess of DFO. E. Lindmo binding assay results from the Xn-XptA2 YWKT construct after DFO conjugation using 8-fold molar excess DFO and Zirconium-89 radiolabeling. F. Lindmo binding assay results from the Xn-XptA2 wt after DFO conjugation using 8-fold molar excess DFO and Zirconium-89 radiolabeling.

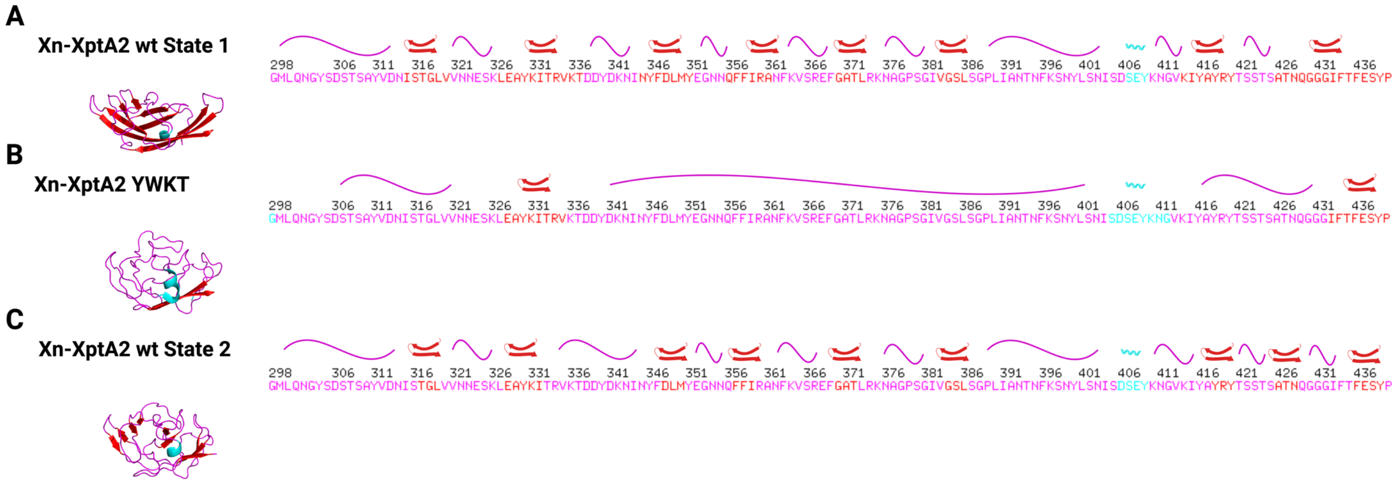

Figure S2. Comparison of Secondary Structure in Xn-XptA2 RBD A. A. Structural representation of Xn-XptA2 wt RBD A in State 1 followed by the amino acid sequence with secondary structure represented above each span of sequence area. B. Structural representation of Xn-XptA2 YWKT RBD A in State 2 followed by the amino acid sequence with secondary structure represented above each span of sequence area. C. Structural representation of Xn-XptA2 wt RBD A in State 2 followed by the amino acid sequence with secondary structure represented above each span of sequence area.

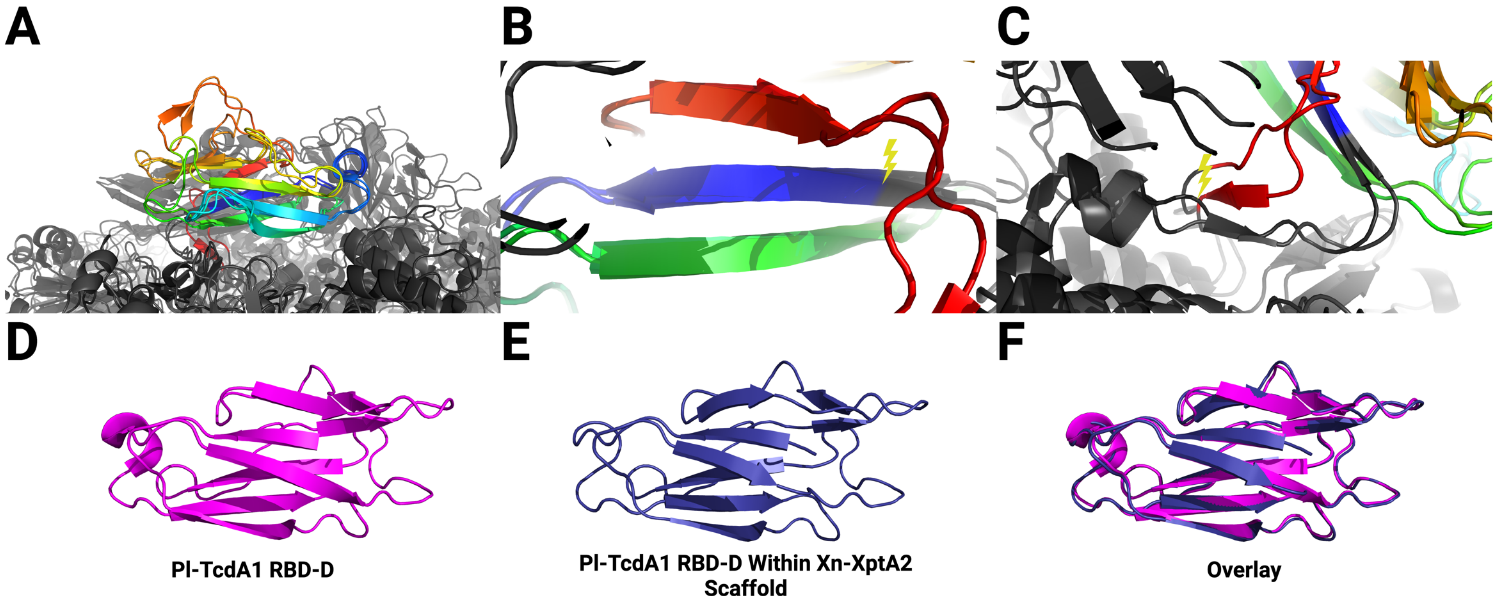

Figure S3. Xn-XptA2 RBD C Chimera Overview. A. Xn-XptA2 and Pl-TcdA1 RBD C/RBD D overlay colored using chainbow. B. Zoomed view of the Xn-XptA2 and Pl-TcdA1 RBD C/RBD D overlay colored using chainbow with a lightning bolt denoting the start of the excision of RBD C/D representing residue F1569 and A1643 respectively. C. Zoomed view of the Xn-XptA2 and Pl-TcdA1 RBD C/RBD D overlay colored using chainbow with a lightning bolt denoting the end of the excision of RBD C/D representing residue G1787 and N1752 respectively. D. Structure of Pl-TcdA1 RBD D (Magenta). E. Structure of Pl-TcdA1 RBD D within the Xn-XptA2 RBD chimera structure (Blue). F. Overlay displaying Pl-TcdA1 RBD D wt (magenta) overlaid with Pl-TcdA1 RBD D within the Xn-XptA2 RBD chimera (Blue).

	Xn-XptA2 wt State 2
Microscope	FEI Polara
Voltage (kV)	300
Camera	K2
Energy filter	none
Physical pixel size	1.268
Defocus range (μm)	-0.8 to -2.5
**Cryo-EM Data and Map**	
Number of micrographs	1605
Number of particles (3D refinement)	42,561
Resolution (dFSC half maps; 0.143, Å)	3.8
Sharpening B-factor ($Å^2$)	173
**Model vs. Map**	
CC (mask)	0.74
CC (peak)	0.67
CC (volume)	0.74
d FSC model (0.5, Å)	3.9
**Model**	
Number of chains	5
Number of protein atoms	12685
RMSD bond length (Å)	0.006
RMSD bond angles	0.830
Molprobity Score	-1.15
All atom clash score	18.33
Ramachandran Favored (%)	91.84
Ramachandran Allowed (%)	8.02
Ramachandran Outliers (%)	0.14
Rotamer Outliers (%)	0
Cβ outliers (%)	0

Table S1. Cryo-EM data collection and refinement statistics for wild type Xn-XptA2.

	Xn-XptA2 YWKT	Xn-XptA2 RBD C Chimera
Microscope	Jeol JEM3200	Titan Krios
Voltage (kV)	300	300
Camera	K2	K3
Energy filter	No	Yes
Physical pixel size	0.642	0.4495
Defocus range (μm)	-0.5 to -3	-0.5 to -2.5
**Cryo-EM Data and Map**		
Number of micrographs	636	2,213
Number of particles (3D refinement)	62,247	77,025
Resolution (dFSC half maps; 0.143, Å)	4.3	3.6
Sharpening B-factor (Å²)	160.7	136.2
**Model vs. Map**		
CC (mask)	0.87	0.89
CC (peak)	0.84	0.86
CC (volume)	0.87	0.89
d FSC model (0.5, Å)	4.3	3.4
**Model**		
Number of chains	5	5
Number of protein atoms	12700	12595
RMSD bond length (Å)	0.002	0.002
RMSD bond angles	0.627	0.529
Molprobity Score	-0.26	1.25
All atom clash score	11.04	4.54
Ramachandran Favored (%)	93.54	95.62
Ramachandran Allowed (%)	6.30	4.14
Ramachandran Outliers (%)	0.16	0.24
Rotamer Outliers (%)	0.05	0.55
C**β** outliers (%)	0	0

Table S2. Cryo-EM data collection and refinement statistics for both Xn-XptA2 YWKT and Xn-XptA2 RBD C Chimera.

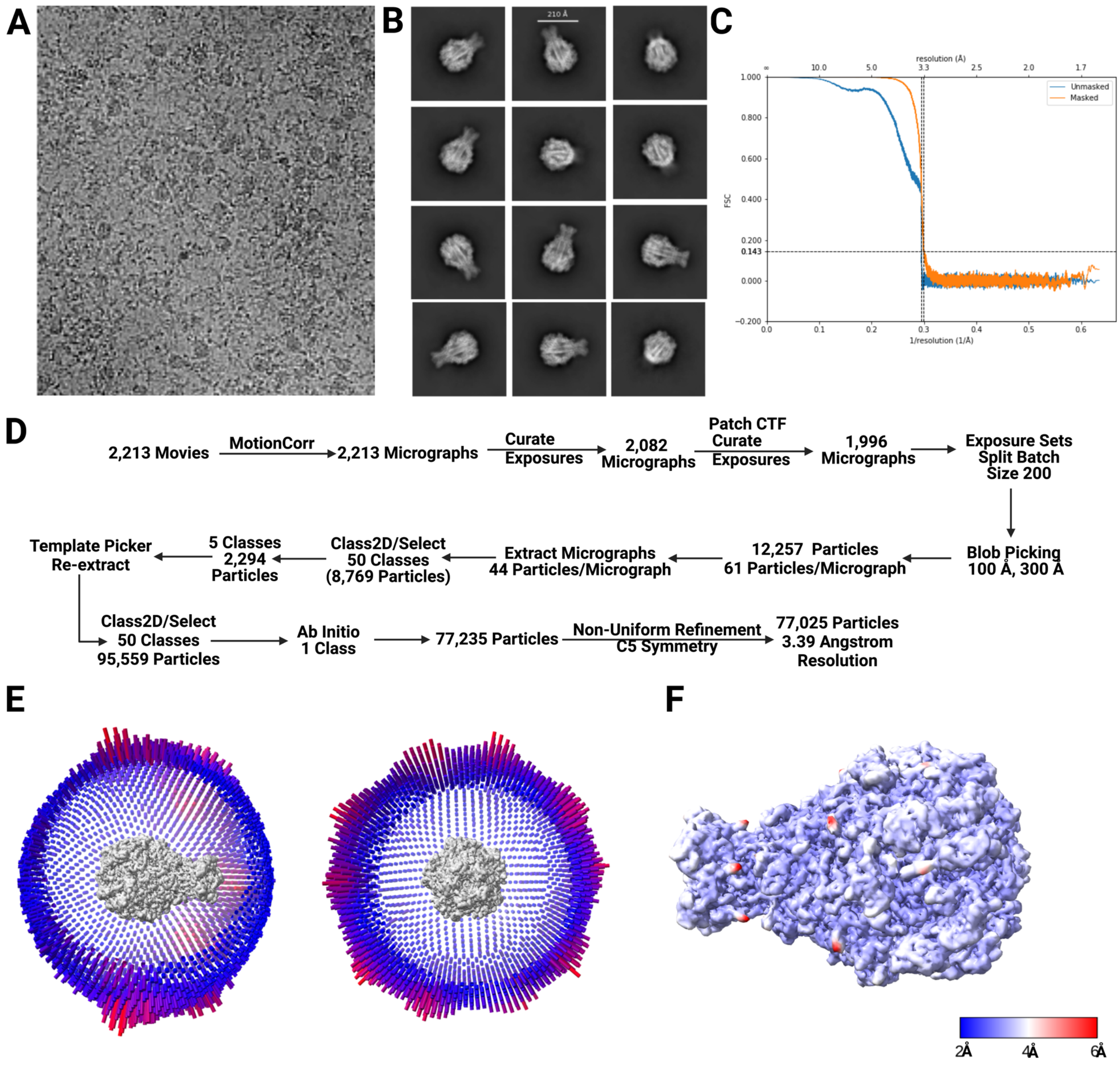

Figure S4. Cryo-EM processing and analysis of Xn-XptA2 RBD C Chimera.

A. Representative cryo-EM micrograph.

B. Representative cryo-EM 2D class averages.

C. FSC curves of the half maps unmasked (blue) versus the masked map (orange).

D. A flowchart for the cryo-EM data processing and structure determination.

E. Euler angle distribution of the final 3D refinement of the overall map.

F. Local resolution map calculated using CryoSPARC.

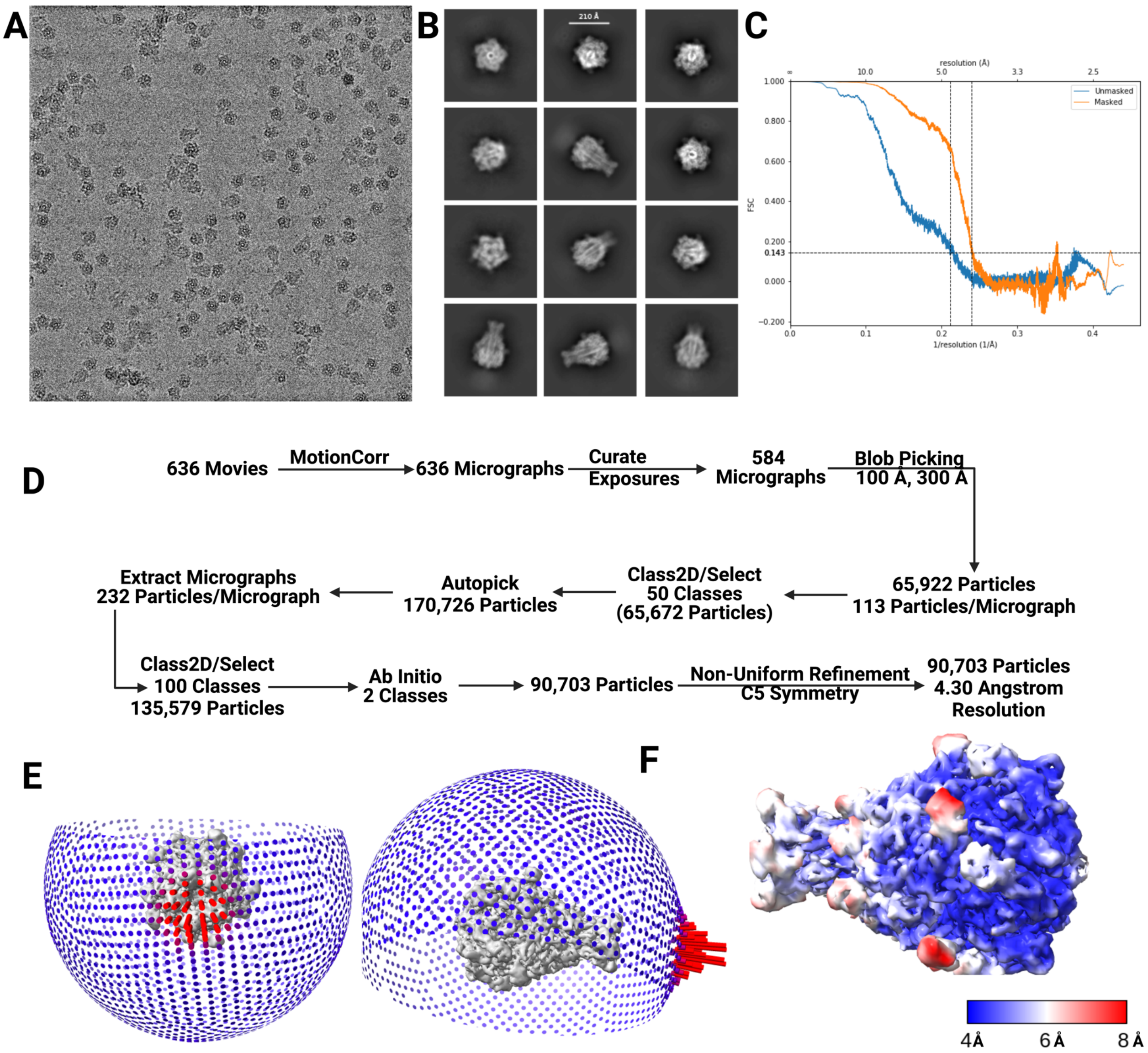

Figure S5. Cryo-EM processing and analysis of Xn-XptA2 YWKT Chimera.
A. Representative cryo-EM micrograph.
B. Representative cryo-EM 2D class averages.
C. FSC curves of the half maps unmasked (blue) versus the masked map (orange).
D. A flowchart for the cryo-EM data processing and structure determination.
E. Euler angle distribution of the final 3D refinement of the overall map.
F. Local resolution map calculated using CryoSPARC.

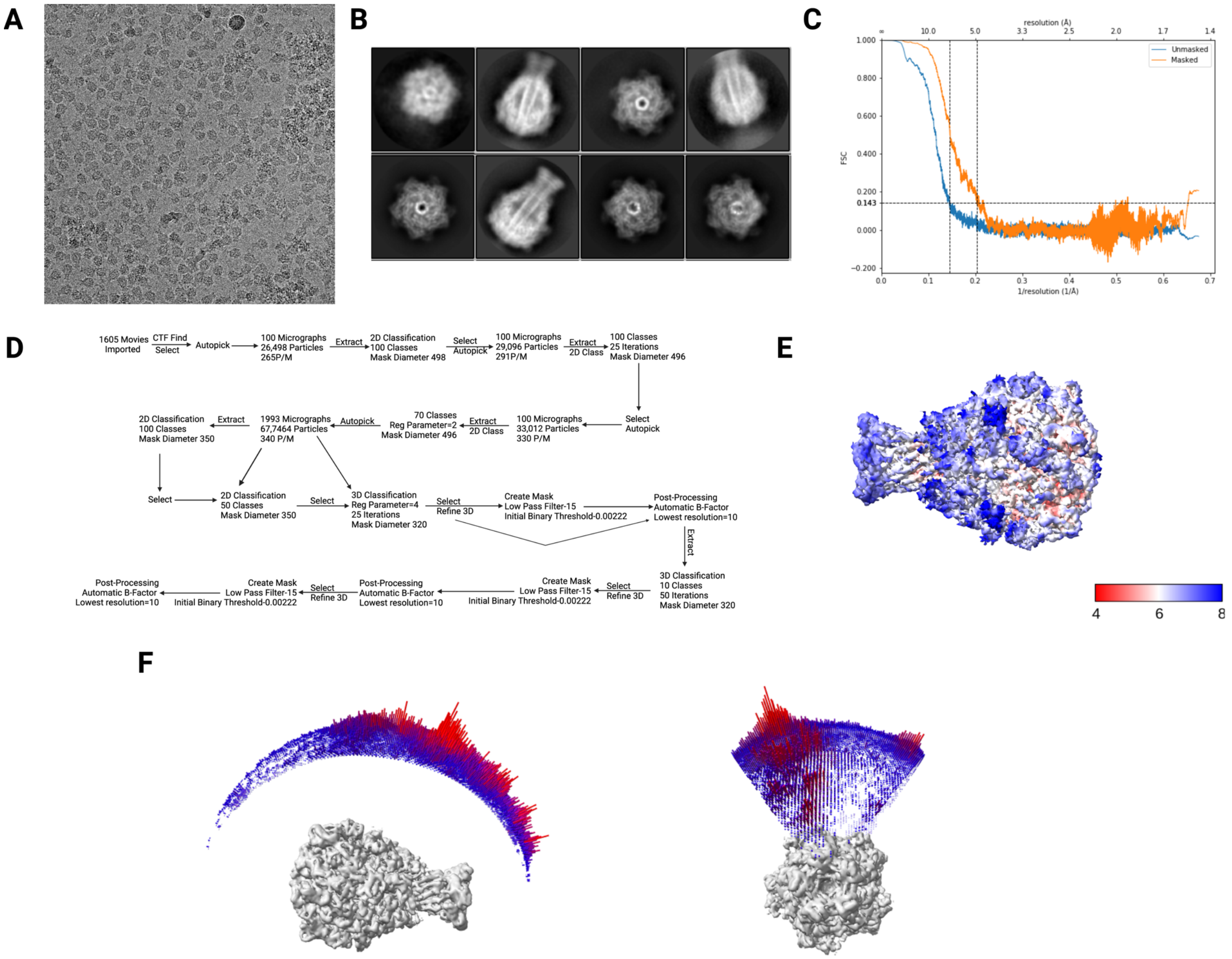

Figure S6. Cryo-EM processing and analysis of Xn-XptA2 wt State 2.

A. Representative cryo-EM micrograph.

B. Representative cryo-EM 2D class averages.

C. FSC curves of the half maps unmasked (blue) versus the masked map (orange).

D. A flowchart for the cryo-EM data processing and structure determination.

E. Local resolution map calculated using CryoSPARC

F. Euler angle distribution of the final 3D refinement of the overall map.

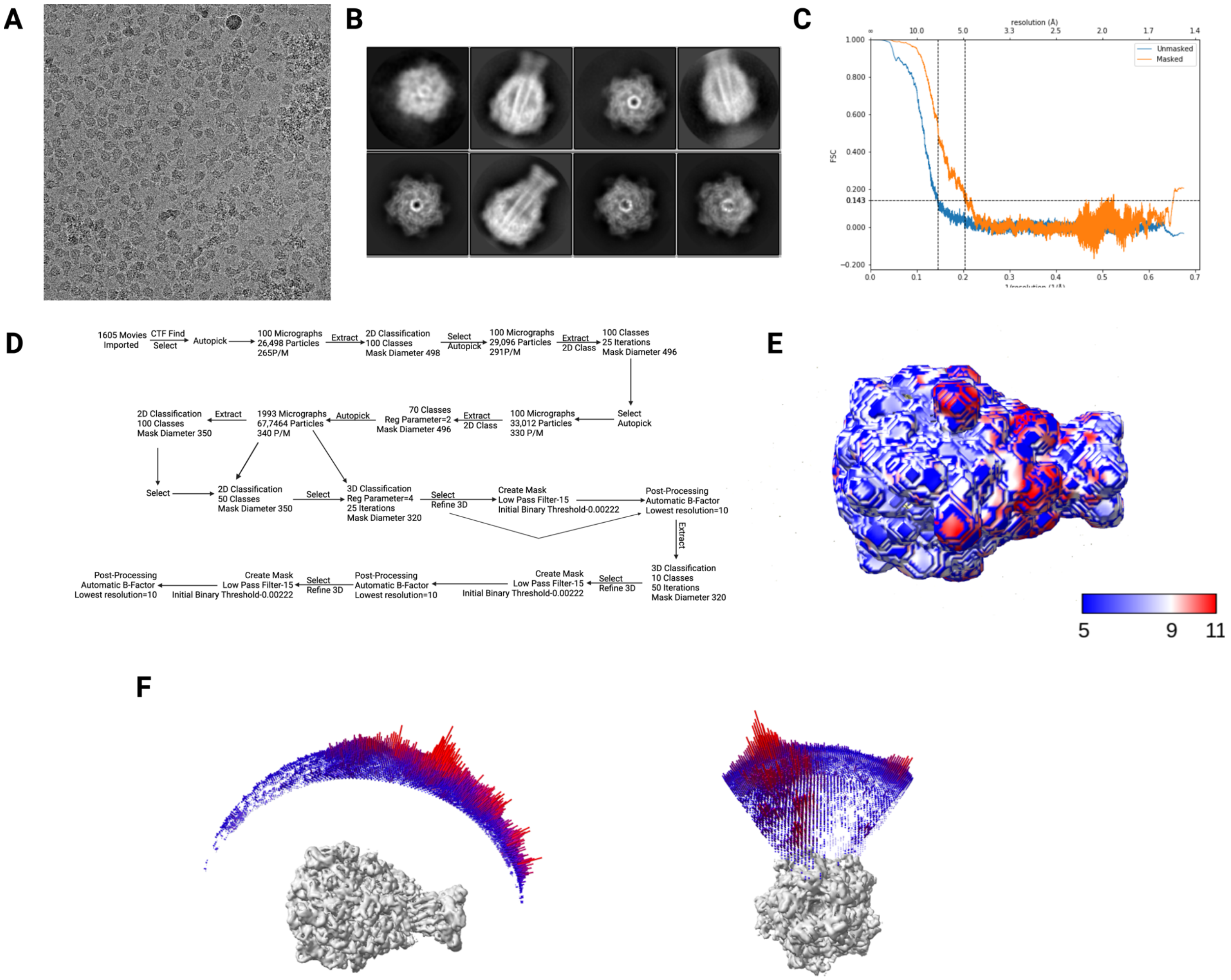

Figure S7. Cryo-EM processing and analysis of Xn-XptA2 State 2 wt.
A. Representative cryo-EM micrograph.
B. Representative cryo-EM 2D class averages.
C. FSC curves of the half maps unmasked (blue) versus the masked map (orange).
D. A flowchart for the cryo-EM data processing and structure determination.
E. Euler angle distribution of the final 3D refinement of the overall map.
F. Local resolution map calculated using Cryosparc.
